# Evaluating *Populus tremula* L. and *Salix caprea* L. for phytoremediation: growth, metal uptake, and biochemical responses under arsenic, cadmium, and lead stress

**DOI:** 10.3389/fpls.2025.1617432

**Published:** 2025-08-01

**Authors:** Vaida Sirgedaitė-Šėžienė, Greta Striganavičiūtė, Milana Šilanskienė, Inesa Kniuipytė, Marius Praspaliauskas, Irena Vaškevičienė, Egidijus Lemanas, Dorotėja Vaitiekūnaitė

**Affiliations:** ^1^ Laboratory of Forest Plant Biotechnology, Institute of Forestry, Lithuanian Research Centre for Agriculture and Forestry, Girionys, Lithuania; ^2^ Laboratory of Heat–Equipment Research and Testing, Lithuanian Energy Institute, Kaunas, Lithuania

**Keywords:** aspen, willow, heavy metals, hydroponics, *hormesis*

## Abstract

This study investigates the phytoremediation potential of *Populus tremula* L. and *Salix caprea* L. in response to As, Cd, and Pb exposure using hydroponics. Seedlings were exposed to 5–50 µM Cd, 100–1000 µM As, and 50–200 µM Pb in *P. tremula*, and to 5–50 µM Cd, 25–100 µM As, and 200–600 µM Pb in *S. caprea*. By analyzing growth, heavy metal(loid) (hereafter referred to as ‘metals’) uptake, biochemical markers (phenolics, soluble sugars, lipid peroxidation, antioxidant enzymes), and shifts in elemental (P, S, Mg, K, Ca, Mn, Zn, Cu, Fe) composition, this study provides a comprehensive evaluation of these species response to metal contamination at the seedling stage. Distinct dose- and metal-specific responses were observed, with Pb exposure inducing enhancing growth effects (height increase up to 27%, total chlorophyll increase up to 67%) and hormesis at low to moderate concentrations (equivalent to 200 µM of Pb(NO_3_)_2_), while Cd and As reduced growth in both species. Biochemical analyses revealed significant impacts on the antioxidant activity in response to metal stress, with differences in the involvement of enzymatic vs. non-enzymatic defenses, i.e., an initial enzymatic response, and a shift towards secondary metabolite production under prolonged or severe stress. *S. caprea* exhibited higher translocation of Cd (0.77 at 5 µM), suggesting its potential for phytoextraction, while both species demonstrated strong phytostabilization capacity for Pb (up to 0.54% of Pb in root DW). Nutrient homeostasis disruptions were observed, with both species showing altered nutrient uptake and distribution, e.g., co-accumulation of Cd and Zn, with Zn increase up to 639% in Cd-treated *S. caprea* (50 µM). These results offer valuable insights into the biochemical mechanisms underlying heavy metal tolerance in *P. tremula* and *S. caprea*, while suggesting directions for future studies on the real-world applicability of phytoremediation strategies.

## 1 Introduction

Heavy metal(loid)s (HMs) (hereafter referred to as ‘metals’) such as cadmium (Cd), arsenic (As), and lead (Pb) are persistent environmental contaminants that cannot be metabolically degraded, and thus can accumulate in ecosystems, leading to detrimental effects on plants, animals, and ultimately human health (De Vries et al., 2022; Sharma et al., 2023). In Europe, efforts to reduce HM emissions have been implemented, resulting in notable decreases between 2005 and 2022: Pb emissions decreased by 44%, and Cd by 39% across the EU-27 Member States (De Vries et al., 2022). However, despite these reductions, HMs continue to accumulate in ecosystems, posing ongoing risks. As an example, the spatial distribution of Cd in the topsoil across the EU and the United Kingdom was analyzed using 21,682 soil samples from the LUCAS soil module, collected in 2009. Among these samples, 5.5% contained Cd concentrations exceeding 1 mg/kg (Ballabio et al., 2024) while a total of at least 10% (depending on the imposed limits, 19 or even 36%) of all tested agricultural soil samples exceeded the limits for the tested HMs (As and Pb included) (Yunta et al., 2024). Based on these results, the EU soil strategy states that by 2050, soil pollution should be reduced to levels no longer considered harmful to human health and the environment (European Commission (EC), 2021).

Phytoremediation has emerged as a sustainable and ecologically sound approach to mitigate HM contamination long term. This green technology utilizes the natural capabilities of plants to remove, stabilize, degrade, or sequester pollutants from contaminated soils. Compared to traditional physical and chemical methods, phytoremediation is generally more cost-effective, particularly in large-scale applications, as it requires minimal input once the plants are established. It’s also less environmentally disruptive, in fact, it can enhance the ecological balance by promoting plant growth and biodiversity (Kumar et al., 2022; Sharma et al., 2023).

Among the different phytoremediation strategies, phytoextraction - metal uptake and translocation to harvestable shoots - and phytostabilization - metal retention in roots or the rhizosphere - are particularly relevant to woody species. The efficiency of either mechanism depends on plant traits, pollutant type, and environmental conditions (Kumar et al., 2022), and directly informs the selection of species for either metal removal or containment. Despite the promise of phytoremediation, several challenges impede its widespread application. The process can be slow, often requiring multiple growing seasons to achieve meaningful pollutant removal. Additionally, the disposal of contaminated plant biomass must be managed carefully to prevent secondary contamination. The efficiency of phytoremediation is influenced by site-specific factors, such as soil and site properties, pH, contaminant type, moisture levels, and temperature, which vary widely across locations (Fendrich et al., 2024; Yunta et al., 2024). Moreover, it has been shown that the capacity for HM accumulation varies greatly among species and even among cultivars within the same species (Kumar et al., 2022; Sharma et al., 2023), hence selecting appropriate plant species with high contaminant uptake and tolerance is critical, yet only a limited number of species possess these capabilities.

A notable research gap exists in the identification and utilization of plant species for phytoremediation purposes. In September 2017, a global database cataloged 754 hyperaccumulator plant species, including 5 As hyperaccumulators, 7 for Cd, and 8 for Pb, among others (Reeves et al., 2017). Hyperaccumulators are plants capable of absorbing and accumulating exceptionally high concentrations of HMs or other contaminants in their tissues without suffering toxic effects. The majority of hyperaccumulator research has focused on herbaceous plants (Rascio and Navari-Izzo, 2011; Kumar et al., 2022), with limited studies on trees or other woody species. Trees, particularly fast-growing species, offer advantages such as extensive root systems and substantial biomass production, which could enhance phytoremediation efficiency. Few tree species have been studied for their phytoremediation potential, and even fewer have been identified as hyperaccumulators (Rascio and Navari-Izzo, 2011; Reeves et al., 2017). Nonetheless, several studies have already demonstrated the potential of *Salix* and *Populus* species for heavy-metal remediation (Konlechner et al., 2013; Salam et al., 2022). However, most of them focused on cuttings or field studies with a notable gap in researching early seedling establishment and response to contaminated environment.

To address this gap, the present study aims to evaluate the effectiveness of two fast-growing tree species, *Populus tremula* L. (European aspen) and *Salix caprea* L. (goat willow), in remediating Cd, Pb, and As contaminated substrates at an early developmental stage. This research provides a novel contribution by systematically comparing the responses of these two ecologically important *Salicaceae* species to three distinct HMs (Cd, Pb, As) during the critical early seedling stage, when foundational tolerance mechanisms are established and phytoremediation potential can be assessed. These species were selected based on their widespread distribution across temperate Europe, rapid biomass production, tolerance to poor soil conditions, and prior use in land reclamation. Additionally, the study seeks to determine whether either species functions as a hyperaccumulator of these metals, thereby contributing to the development of more efficient and sustainable phytoremediation strategies. While previous studies have typically focused on mature plants or cuttings, our comprehensive approach enables direct comparison of early-stage metal-specific responses across both species under standardized conditions. Based on the known physiological characteristics of *Salicaceae* species and preliminary studies on related taxa, we hypothesize that: (I) both species will demonstrate greater tolerance to Pb than to Cd or As, (II) *S. caprea* will exhibit higher translocation factors than *P. tremula* due to its documented metal accumulation capabilities, and (III) successful metal tolerance will be accompanied by upregulated antioxidant systems and altered nutrient homeostasis. Our systematic comparative approach using controlled hydroponic conditions will reveal metal-specific bioaccumulation patterns that inform optimal phytoremediation applications for each species and provide essential baseline data for early-intervention phytoremediation strategies.

## 2 Materials and methods

### 2.1 Seeds and initial growth environment

*Populus tremula* L. and *Salix caprea* L. seeds were collected in the spring of 2024 and stored at +4°C until the start of the experiment (2–3 months). The seeds were sprouted in the lab (+22°C) in glass Petri dishes on moist (tap water) filter paper and when roots emerged the seedlings were transferred to moist rockwool cubes (soaked in distilled water, pH 5.6, adjusted using HCl and NaOH) and grown in modified full-strength (100%) Hoagland’s nutrient medium, containing 6 mM KNO_3_, 2.32 mM Ca(NO_3_)_2_×4H_2_O, 1.86 mM MgSO_4_×7H_2_O, 1 mM NH_4_H_2_PO_4_, 46 μM H_3_BO_3_, 9 μM MnCl_2_×4H_2_O, 8.99 μM C_12_H_12_Fe_2_O_18_, 0.76 μM ZnSO_4_×7H_2_O, 0.5 μM CuSO_4_×5H_2_O, and 0.58 μM Na_2_MoO_4_×2H_2_O. The seedlings were incubated under semi-controlled conditions (day/night temperature of 25/20°C, natural light) for 5 weeks until the BBCH-14 growth stage (4 true leaves) was reached (Guilayn et al., 2020). The seedlings were watered as needed with half-strength (50%) Hoagland’s nutrient medium.

### 2.2 Hydroponic conditions and experimental groups

After the BBCH-14 growth stage was reached, the seedlings were transferred to the hydroponic system into 12 L non-see-through plastic containers (42×35×11 cm). The container covers had holes with plastic aquatic pots. Each container was filled with 8 L of full-strength Hoagland’s medium and the rockwool cubes were placed into the aquatic pots with the lower half fully submerged. For both *P. tremula* and *S. caprea* 10 experimental groups were studied: control group and 9 treatment groups. The medium in 9 containers was contaminated with HMs – As (AsH_15_Na_2_O_11_, 98% purity; Thermo Fisher, Germany), Pb (Pb(NO_3_)_2_, 99% purity; VWR International GmbH, Austria) and Cd (CdSO_4_, 99% purity; Thermo Fisher) right before transferring the seedlings. For *P. tremula* the 9 groups were 3 concentrations of As (100, 500 and 1000 µM), 3 concentrations of Pb (50, 100 and 200 µM) and 3 concentrations of Cd (5, 25, 50 µM). For *S. caprea* the 9 groups were treated with 3 concentrations of As (25, 50, 100 µM), 3 concentrations of Pb (200, 400 and 600 µM) and 3 concentrations of Cd (5, 25, 50 µM). Continuous aeration was provided by air pumps (14020; 220 V, 50 Hz, 5 W, China) connected to plastic tracheas, and an oxygen diffuser (18 cm) secured to the bottom of the container. The seedlings were grown under semi-controlled conditions (day/night temperature of 25/20°C, natural light) for 4 weeks in a greenhouse. During this time the seedlings were watered as needed with full-strength Hoagland’s medium.

### 2.3 Growth measurements and sampling

Immediately after transferring to the hydroponic conditions, seedling height was measured. This measurement was repeated after 4 weeks to determine height increase over the period. The longest root length was only measured at the end of the experiment, as root systems were embedded in rockwool cubes and could not be measured accurately without disrupting growth. Tolerance index (TI) was calculated based on modified formula from shoot height and shows a relative measure of a plant’s ability to withstand and grow in the presence of contaminants (Yongpisanphop et al., 2017). The aboveground and root biomass of the seedlings were collected separately for subsequent biochemical and elemental composition analyses. For the biochemical analyses, 6 fresh samples of 0.1 g were taken from the leaf and 6 from the root tissues from each experimental group. Each sample was comprised of smaller samples taken from the biomass of the whole group. The samples were frozen for subsequent analyses. For the elemental composition analyses, the root and shoot biomass was completely dried at 60°C in a forced-air ventilation oven (Binder FD 115, Germany) to a constant dry weight (DW) and weighed again (Mettler Toledo XP2003S DR, Switzerland). Three samples of ~0.2-0.4 g were taken from each experimental group.

### 2.4 Biochemical analyses

Seedling total chlorophyll, carotenoid (CAR), secondary metabolite (SM): total phenol (TPC) and total flavonoid (TFC), lipid peroxidation level, i.e., malondialdehyde (MDA), soluble sugar, (SS), antioxidant enzyme (catalase (CAT), superoxide dismutase (SOD), guaiacol peroxidase (POX), ascorbate peroxidase (APX), glutathione reductase (GR), glutathione S-transferase (GST)) levels were measured spectrophotometrically using a SpectroStar Nano microplate reader (BMG Labtech, Offenburg, Germany) with 96-well microplates. A detailed list of the formulas for biochemical analyses done are listed in the relevant references and **Supplementary Table 1**.

#### 2.4.1 Extract preparation for photosynthetic pigment, secondary metabolite, MDA, and sugar analysis

For each experimental group, three 0.1 g samples were pulverized using a tissue homogenizer (Precellys 24, Bertin Technologies, France) at 1956×g for 30 seconds with the addition of two metal beads. Next, 1.5 mL of 80% ethanol (v/v in water, MV GROUP Production, Lithuania) was added, and the homogenization process was repeated under the same conditions. The resulting mixture was then centrifuged at 16,090×g for 30 minutes at 4°C using a Hettich Universal 32R centrifuge (Andreas Hettich GmbH & Co. KG, Germany). The supernatant was collected and used for the analysis of total chlorophyll, CAR, TPC, TFC, MDA, and SS.

#### 2.4.2 Total phenol content

In brief, the extract was mixed with Folin–Ciocalteu reagent (VWR International GmbH) (1:9 v/v in water) and incubated for 5 minutes. Subsequently, 10% Na_2_CO_3_ was added, and the mixture was kept in the dark for 1 hour. Absorbance was then measured at 725 nm. The formulas and calibration for the analysis were described in detail previously (Beniušytė et al., 2023).

#### 2.4.3 Total flavonoid content

Briefly, for TFC the extract was combined with a reaction buffer containing absolute ethyl alcohol (MERCK, Germany), aluminum chloride (Alfa Aesar, Germany), potassium acetate (Sigma Aldrich, USA), and distilled water (dH_2_O). The mixture was incubated in the dark for 30 minutes. Absorbance measurements were taken at 415 nm. The formulas and calibration for the analysis were as described in detail previously (Beniušytė et al., 2023).

#### 2.4.4 Chlorophyll *a* and *b*, and carotenoid content

Absorbance of the extract was measured at 470, 648, and 664 nm. The formulas and calibration for the analyses were as described in detail previously (Beniušytė et al., 2023). Total chlorophyll content was calculated by adding chlorophyll a and b content.

#### 2.4.5 Malondialdehyde content

In brief, MDA was quantified using a modified method. The supernatant was mixed with a reaction mixture containing trichloroacetic acid (Molar Chemicals Kft, Hungary) and thiobarbituric acid (Alfa Aesar). The mixture was incubated at 95°C for 30 minutes and then cooled on ice. Absorbance was recorded at 440, 532, and 600 nm. The formulas and further details for the analysis were described previously (Čėsnienė et al., 2023).

#### 2.4.6 Soluble sugar content

Soluble sugars were quantified by mixing the sample with anthrone reagent (Carl Roth, Germany), prepared by dissolving anthrone in concentrated H_2_SO_4_ (Chempur, Poland) according to Čėsnienė et al (Čėsnienė et al., 2023). The mixture was incubated at 90°C for 1 hour, and absorbance was measured at 620 nm.

#### 2.4.7 Potassium phosphate buffer preparation

K-phosphate buffers of varying pH values were prepared by mixing stock solutions of 1 M K_2_HPO_4_ (Carl Roth) and 1 M KH_2_PO_4_ (Chempur). Buffers with pH values of 6.5, 7.0, 7.4 and 7.8 were prepared as required.

#### 2.4.8 Extract preparation for enzyme analysis

For enzyme activity assays, three 0.1 g samples of fresh biomass from each experimental group were pulverized in liquid nitrogen using a mortar and pestle and then mixed with an extraction buffer containing K-phosphate buffer (pH 7.8), Triton X (Carl Roth), polyvinylpolypyrrolidone (PVPP) (Sigma Aldrich), and ascorbic acid (ASC) (Chempur). The mixture was centrifuged at 14,020×*g* at −4°C for 1 hour, and the resulting supernatant was used to measure protein levels, as well as CAT and SOD activity. For APX, POX, GR, and GST assays, the supernatant was further purified using Sephadex G-25 columns (Column PD-10, Cytiva, UK), and the bulk filtrate was used for the latter analyses. All procedures were carried out on ice to preserve sample integrity. The formulas and further details for the analysis were described previously (Beniušytė et al., 2023).

#### 2.4.9 Total protein quantification

Protein concentration was determined by mixing the crude extract with Biuret reagent, composed of CuSO_4_, Na-K tartrate (Eurochemicals, Lithuania), and Na_2_CO_3_ in NaOH solution, followed by the addition of Folin–Ciocalteu reagent. After incubating for 50 minutes at room temperature, absorbance was measured at 660 nm. The formulas and further details for the analysis were described previously (Beniušytė et al., 2023).

#### 2.4.10 Catalase activity

Catalase activity was assessed by mixing the crude extract with K-phosphate buffer (pH 7) and 30% H_2_O_2_. Absorbance was measured at 240 nm at regular intervals to monitor the activity. The formulas and further details for the analysis were described previously (Beniušytė et al., 2023).

#### 2.4.11 Superoxide dismutase activity

SOD activity was assessed by mixing the crude extract with a reaction buffer containing K-phosphate buffer (pH 7.8), methionine (AppliChem, Germany), nitro blue tetrazolium (NBT) (VWR International GmbH), ethylenediaminetetraacetic acid (EDTA) (Chempur), and riboflavin (AppliChem). The reaction mixture was exposed to white light (irradiance 30 μmol m^-^² s^-^¹) until the samples darkened relative to the control. Absorbance was then measured at 550 nm to determine SOD activity. The formulas and further details for the analysis were described previously (Čėsnienė et al., 2023).

#### 2.4.12 Guaiacol peroxidase activity

POX activity was evaluated by mixing the filtered extract with a reaction solution containing pyrogallol (Chempur), a 50 mM K-phosphate buffer (pH 6.5), and 30% H_2_O_2_. Absorbance at 430 nm was measured at regular intervals to monitor the activity. The formulas and further details for the analysis were described previously (Beniušytė et al., 2023).

#### 2.4.13 Ascorbate peroxidase activity

APX activity was measured by mixing the filtered extract with ascorbic acid (ASC) solution, 50 mM K-phosphate buffer (pH 7.0), and 30% H_2_O_2_. Absorbance at 290 nm was recorded at regular intervals to monitor the activity. The formulas and further details for the analysis were described previously (Beniušytė et al., 2023).

#### 2.4.14 Glutathione S-transferase activity

GST activity was measured by combining the filtered extract with a reaction buffer containing K-phosphate buffer (pH 6.5), 1-chloro-2,4-dinitrobenzene (CDNB) (Thermo Fisher), and reduced L-glutathione (GSH) solution (Sigma Aldrich). Absorbance at 340 nm was recorded at regular intervals to monitor the activity. The formulas and further details for the analysis were described previously (Sirgedaitė−Šėžienė et al., 2024).

#### 2.4.15 Glutathione reductase activity

To measure GR enzyme activity, the filtered extract was combined with a reaction buffer consisting of HEPES buffer (pH 8) (Sigma Aldrich), EDTA, and NADPH (Carl Roth). After adding oxidized L-glutathione (Applichem) (GSSG), absorbance changes at 340 nm were monitored at regular intervals. The formulas and further details for the analysis were described previously (Beniušytė et al., 2023).

### 2.5 Elemental composition analyses

The previously dried samples were pulverized using an IKA MF 10 mill (IKA^®^-Werke GmbH & Co. KG, Germany). The pulverized samples were then digested with a mixture of 65% HNO_3_, 30% H_2_O_2_, and 48% HF in a volume ratio of 8:3:1. The digestion process was performed using a high-pressure microwave digester (Anton Paar, Multiwave GO Plus, Austria). The concentrations of Cd, As, Pb, P, S, Mg, K, Ca, Mn, Zn, Cu, and Fe in the digested shoot and root samples were determined using inductively coupled plasma optical emission spectrometry (ICP-OES, Optima 8000, PerkinElmer, USA). To ensure the accuracy of the analytical methods certified reference material (CRM) was used, specifically CRM-BCR-129 hay powder, approved by the Community Bureau of Reference (BCR). To monitor ICP-OES signal drift, a control solution (5 mg/L) was analyzed every 20 samples. Calibration was repeated whenever the measured value deviated by more than 10% from the predetermined boundaries. Each sample was analyzed in triplicate to ensure accuracy and reproducibility. Element calibration was conducted at four calibration points using PerkinElmer standard solutions: for Cd, As, Pb, Mg, Ca, Mn, Zn, and Fe, the Multi-Element Quality Control Standard 21 was used; for K, the Quality Control Standard 7A was used; and for P and S, Pure Standards were used. The accuracy of elemental analysis was evaluated using a linear correlation coefficient of at least 0.999, ensuring high precision in the results (Kniuipytė et al., 2023).

Additionally, in response to HM exposure, several indices were calculated following the formulas provided by Yongpisanphop et al (Yongpisanphop et al., 2017): bioconcentration factor (BCF) for tree leaves and roots showing how much HM was found in these tissues as compared to the nutrient medium and translocation factor (TF) showing how much of the HMs was transferred from roots to leaves. A detailed list of the formulas for the indices used are listed in the relevant references and **Supplementary Table 1**.

### 2.6 Statistical analysis

The data were compiled, and graphs were generated using Microsoft Office Excel. Statistical analyses were conducted with SPSS version 28.0.1.1 (IBM Inc.). Since the biochemical data did not meet normality or homogeneity assumptions, even after transformation, the Kruskal–Wallis H test was used for independent samples. Pairwise rank comparisons were performed using Dunn’s *post hoc* test (p<0.05). A 95% confidence level was maintained, and the results of Dunn’s test were reported with Bonferroni-adjusted p-values to identify specific groups with significant differences. The elemental composition and metal uptake data were expressed as a mean from 3 pooled samples due to constraints in the amount of biomass the plants produced. And thus lacks a robust statistical analysis.

## 3 Results

### 3.1 HM impact on *Populus tremula*

**Figure 1:**
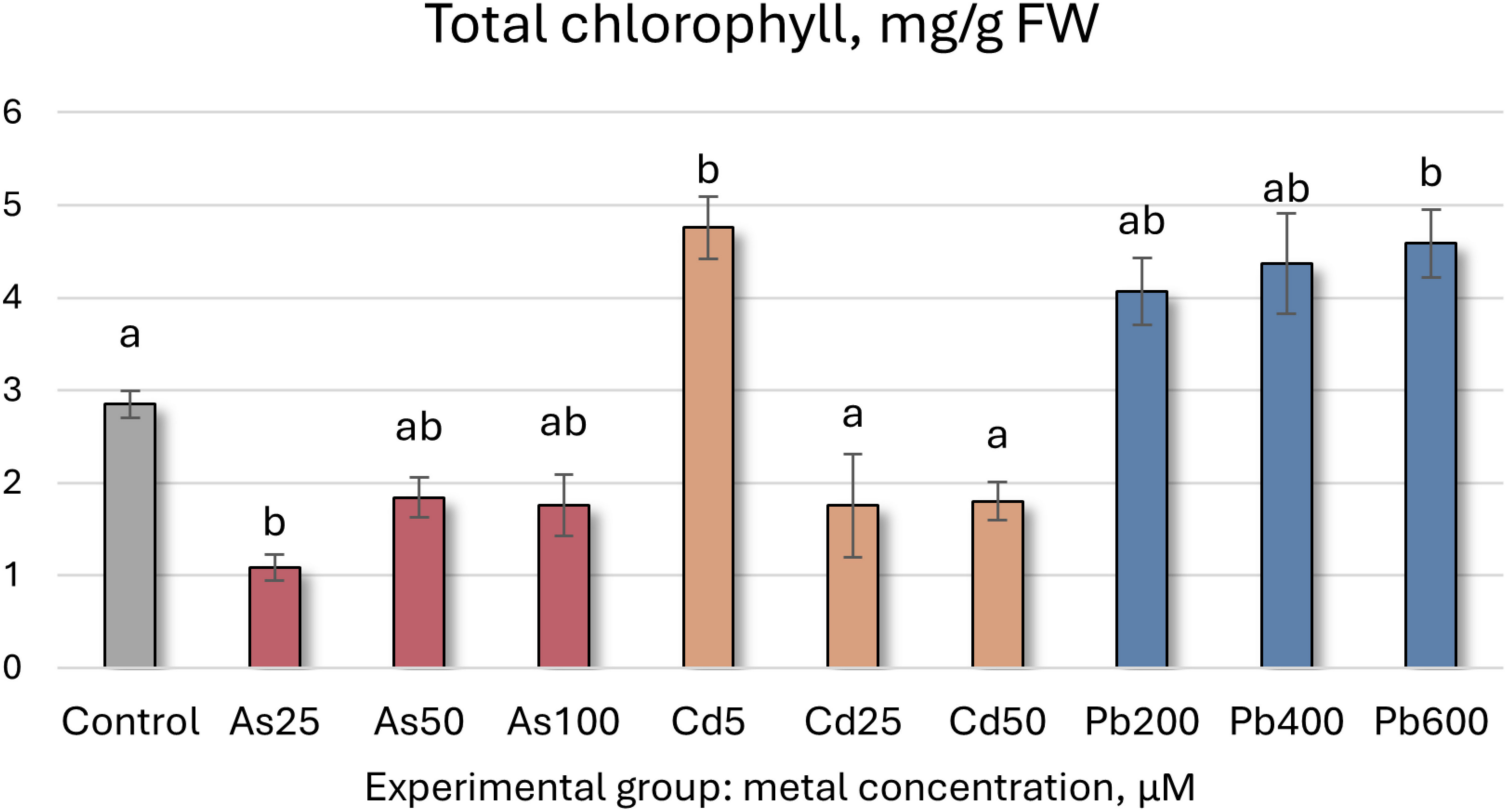
Total chlorophyll concentrations (mg/g FW ± SD) in Salix caprea after 4 weeks grown in hydroponic conditions in different experimental groups (control – untreated, treated with 25, 50, 100 μM of As, 5, 25, 50 μM of Cd, and 200, 400 and 600 μM of Pb). Different letters next to the same color columns and the control denote a significant difference between concentrations based on Dunn’s test (p<0.05).

**Figure 2:**
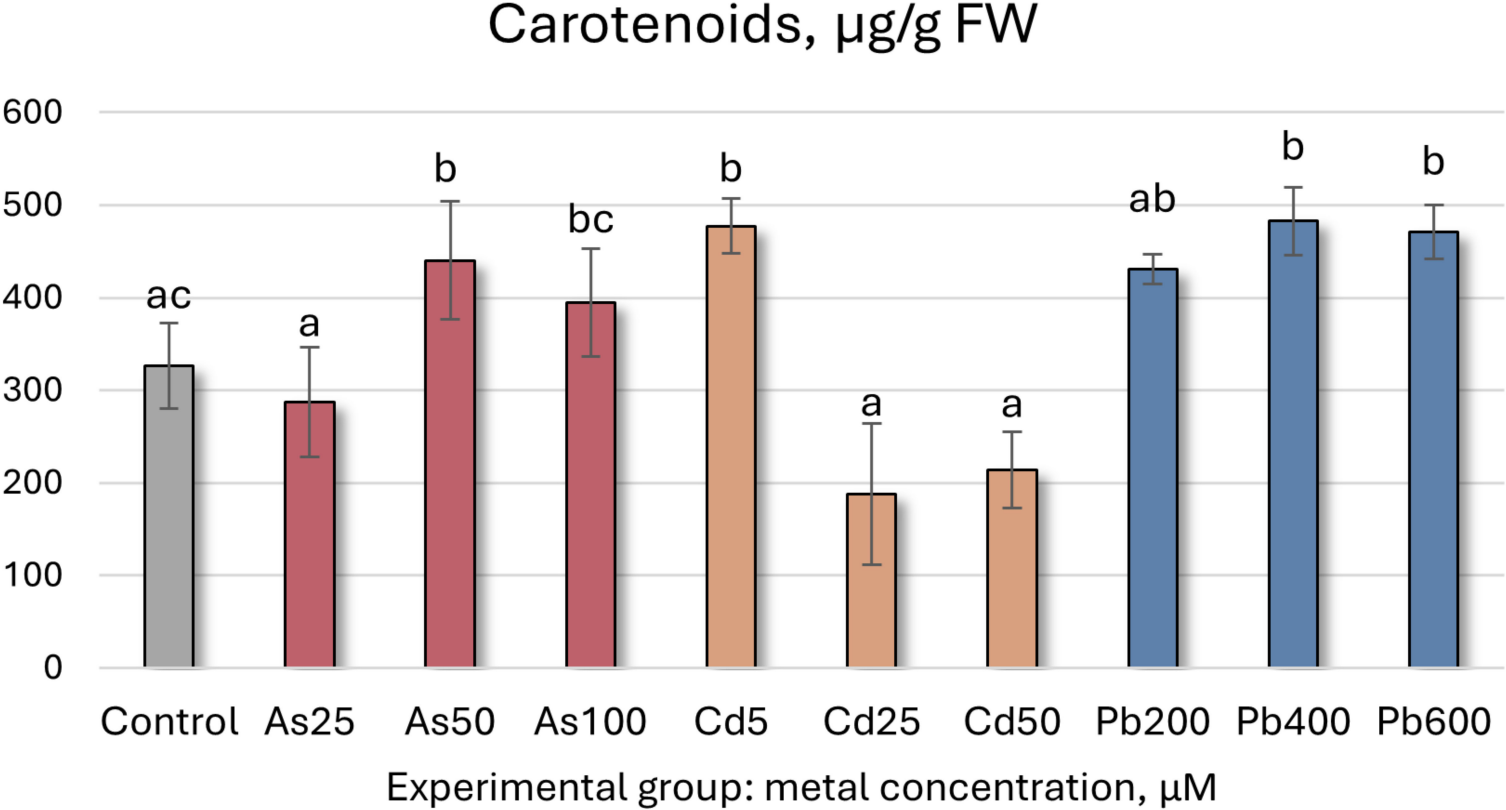
Carotenoid concentrations (μg/g FW ± SD) in Salix caprea after 4 weeks grown in hydroponic conditions in different experimental groups (control – untreated, treated with 25, 50, 100 μM of As, 5, 25, 50 μM of Cd, and 200, 400 and 600 μM of Pb). Different letters next to the same color columns and the control denote a significant difference between concentrations based on Dunn’s test (p<0.05).

Out of the nine treatment groups for *P. tremula*, only seven survived until the end of the experiment. Exposure to 500 and 1000 µM of As resulted in complete shoot growth inhibition and severe chlorosis (**Figure 1**), preventing further analyses for these two groups due to lack of biomass. To assess the seedlings’ ability to tolerate heavy metals (HMs) in the nutrient medium, several indices were calculated for the remaining groups. In all Pb-treated groups, the TI (tolerance index) exceeded 100%, ranging from 113% to 127% (**Table 1**, **Figure 2**), indicating enhanced growth under contamination. The TF (translocation factor) in these groups ranged from 0.01 to 0.06 (**Table 1**). Under Pb exposure, the BCF (bioconcentration factor) for *P. tremula* shoots remained below 1 (0.42–0.84), while in the roots, it ranged from 15.92 to 66.22 (**Table 1**). The precise HM concentrations in leaves and roots for all experimental groups are presented in **Table 1**. Notably, all HM levels increased with rising concentrations in both tissues, with roots consistently exhibiting higher concentrations of the target HMs. Cd levels in leaves ranged from 59 to 538 µg/g DW, while root concentrations varied between 176 and 1713 µg/g DW. In Pb-treated *Populus* seedlings, leaf concentrations ranged from 9 to 17 µg/g DW, whereas root levels were between 165 and 2746 µg/g DW.

**Table 1:** Bioconcentration factor (BCF), tolerance index (TI), translocation factor (TF) and heavy metal (HM) concentrations in the leaves and roots of *Populus tremula* in different experimental groups (from 3 pooled replicates; control – untreated, treated with 100 µM of As, 5, 25, 50 µM of Cd, and 50, 100 and 200 µM of Pb).

HM concentration in medium, µM		BCF		TI (%)	TF	Concentration in biomass, µg/g DW	
		Leaves	Root			Leaves	Roots
As	100	1.52	279.9	69.23	0.01	11.4 ± 1.73	2099.28 ± 96.46
Cd	5	110.25	326.14	81.3	0.34	59.46 ± 12.48	175.88 ± 19.6
	25	104.21	361.14	51.9	0.25	281.01 ± 27.2	973.81 ± 89.84
	50	99.73	317.66	22.62	0.32	537.86 ± 12.48	1713.16 ± 192.19
Pb	50	0.84	15.92	113.36	0.06	8.67 ± 1.1	165.29 ± 61.4
	100	0.8	53.16	114.85	0.02	16.58 ± 2.96	1100.69 ± 264.98
	200	0.42	66.22	127.03	0.01	17.3 ± 4.46	2746.03 ± 624.4

Under Cd exposure, *P. tremula* exhibited low TIs, ranging from 23% to 81% across all tested concentrations (5, 25, and 50 µM), indicating poor seedling health. TFs were relatively high, ranging from 0.25 to 0.34, while BCFs ranged from 99.73 to 110.25 in the leaves and 317.66 to 326.14 in the roots. Similarly, *P. tremula* showed low tolerance to As (100 µM), with a TI of 69% (**Figure 1**, **Table 1**), a TF of 0.01, a low leaf BCF (1.52), and a root BCF of 279.9. In both cases this suggests limited As and Cd translocation but significant root accumulation (**Table 1**).

**Figure 3:**
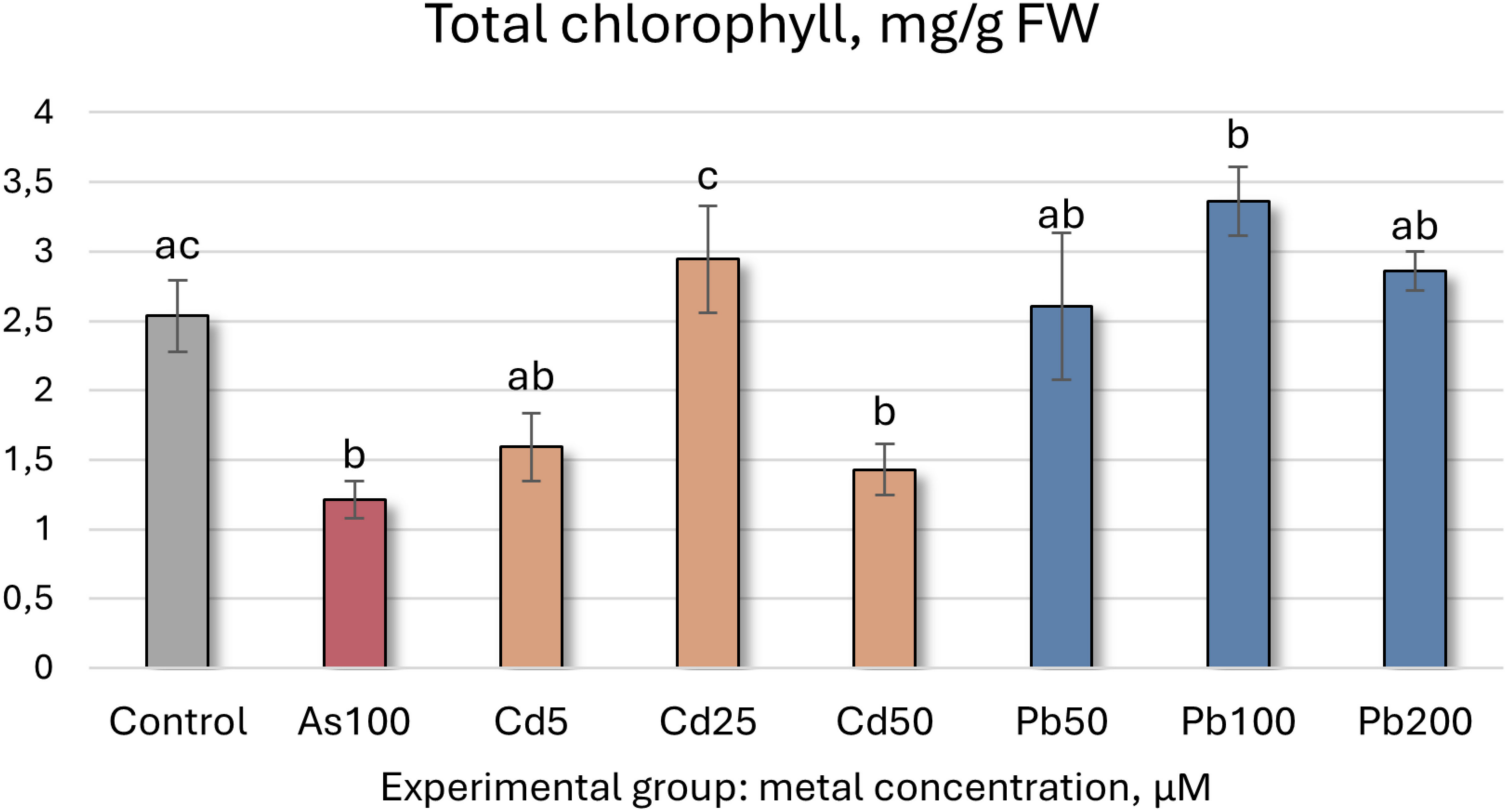
Total chlorophyll concentrations (mg/g FW ± SD) in *Populus tremula* after 4 weeks grown in hydroponic conditions in different experimental groups (control – untreated, treated with 100 µM of As, 5, 25, 50 µM of Cd, and 50, 100 and 200 µM of Pb). Different letters next to the same color columns and the control denote a significant difference between concentrations based on Dunn’s test (*p*<0.05).

HM exposure significantly altered all tested biochemical indicators. While the TI already reflects shoot growth, a closer analysis reveals that As exposure (100 µM) reduced shoot height and root length by 31% and 23%, respectively. In the same group, total chlorophyll concentration decreased by 52% (**Figure 3**), while MDA (indicator for lipid peroxidation and thus cellular damage) and TPC levels increased. TFC, CAR, and SS levels remained stable, whereas antioxidant enzyme activity generally increased, except for POX, which remained unchanged, and SOD, which declined by 34% (**Table 2**). Overall, the interpretation of secondary metabolite, SS, CAR, and antioxidant enzyme data is more complex and should be interpreted holistically, as they can both mean enhanced stress and tolerance.

**Table 2:** Biochemical marker [total chlorophyll, carotenoid (CAR), total phenol (TPC) and total flavonoid (TFC) content, malondialdehyde (MDA), soluble sugar, (SS), antioxidant enzyme (catalase (CAT), superoxide dismutase (SOD), guaiacol peroxidase (POX), ascorbate peroxidase (APX), glutathione reductase (GR), glutathione S-transferase (GST)] levels in the leaves of *Populus tremula* in different experimental groups (control – untreated, treated with 100 µM of As, 5, 25, 50 µM of Cd, and 50, 100 and 200 µM of Pb).

HM concentration in medium, µM	Control	As	Cd			Pb		
	0	100	5	25	50	50	100	200
TPC	0.17 ± 0.01a	0.2 ± 0.03a	0.22 ± 0.03b	0.26 ± 0.01b	0.24 ± 0.01b	0.17 ± 0.02a	0.18 ± 0.01a	0.21 ± 0.03a
TFC	0.21 ± 0.03ab	0.21 ± 0.02a	0.24 ± 0.04a	0.25 ± 0.04a	0.17 ± 0.01b	0.18 ± 0.03a	0.2 ± 0.03a	0.19 ± 0.02a
SS	0.92 ± 0.05a	1 ± 0.14a	0.92 ± 0.11a	1.1 ± 0.39a	1.33 ± 0.17a	0.74 ± 0.08a	0.76 ± 0.07a	0.75 ± 0.09a
MDA	68.78 ± 2.96a	74.14 ± 4.74ab	80.58 ± 4.45b	73.04 ± 5.12ab	71.56 ± 4.91ab	79.53 ± 7.04a	74.77 ± 6.89a	71.57 ± 4.45a
Total chlorophyll	2.53 ± 0.26ac	1.21 ± 0.13b	1.59 ± 0.24ab	2.94 ± 0.38c	1.43 ± 0.18b	2.6 ± 0.53ab	3.36 ± 0.25b	2.86 ± 0.14ab
Carotenoid content	228.4 ± 36.2a	246.53 ± 17.13ab	290.74 ± 47.75b	319.18 ± 42.78b	176.92 ± 43.83a	247.84 ± 54.96a	313.26 ± 17.95b	256.05 ± 26.78a
Shoot height increase	300.6 ± 65.59a	208.11 ± 74.9b	244.4 ± 82.68a	156 ± 40.96b	68 ± 37.66b	340.75 ± 70.45a	345.25 ± 88.26a	381.85 ± 102.49a
Root length	482.1 ± 68.87a	370.21 ± 118.39b	473.55 ± 176.82a	495.4 ± 95.71a	334.1 ± 128.85a	531.75 ± 74.34a	530.5 ± 121.7a	501.2 ± 96.57a
CAT	8.53 ± 0.53a	9.77 ± 1.23b	9.4 ± 0.68b	8.38 ± 0.75a	10.13 ± 1.15b	9.37 ± 1.03b	9.68 ± 1.3b	9.29 ± 0.7ab
APX	92.81 ± 3.53a	119.33 ± 25.87b	102.24 ± 6.94bc	95.77 ± 11.72ab	122.12 ± 23.61c	110.78 ± 20.7b	108.73 ± 8.8b	92.38 ± 6.75a
POX	2.53 ± 0.34a	2.61 ± 0.35a	2.39 ± 0.19a	2.67 ± 0.22a	3.84 ± 0.71b	3.89 ± 0.76b	2.81 ± 0.35ac	3.61 ± 0.78bc
GR	48.49 ± 1.85a	63.24 ± 13.45b	56.89 ± 4.42bc	51.05 ± 5.3ac	67.76 ± 12.65b	60.58 ± 12.02b	60.81 ± 5.33b	56.87 ± 4.17b
GST	25.06 ± 1.24a	29.36 ± 4.07b	27.93 ± 1.94bc	25.7 ± 2.12ac	33.98 ± 7.13b	30.82 ± 5.4b	30 ± 2.33b	28.32 ± 2.12b
SOD	259.39 ± 84.94a	171.03 ± 71.08b	130.61 ± 42.49b	173.31 ± 81.6ab	194.66 ± 91.54ab	221.48 ± 132.85ab	206.07 ± 56.95a	135.36 ± 52.84b

Different letters in the same row for one metal and the control denote a significant difference between concentrations based on Dunn’s test (*p*<0.05).

The data for Cd exposure show similar trends (**Table 2**). Increasing Cd concentrations led to reduced growth, with shoot and root lengths decreasing by 77% and 31%, respectively, at 50 µM. MDA levels showed a slight increase across all Cd treatments. Total chlorophyll content decreased at 5 µM but exhibited a hormesis effect at 25 and 50 µM. Secondary metabolite (TPC and TFC) and CAR levels fluctuated, while antioxidant enzyme activity (CAT, POX, APX, GR, and GST) generally increased, except for SOD, which declined. These findings showcase that both As and Cd are phytotoxic to *P. tremula* at the tested concentrations.

Pb exposure yielded distinct results. Overall, growth remained statistically unchanged, with a slight upward trend as Pb concentrations increased, as can be noted from the TIs (**Tables 1**, **2**; **Figure 2**). Total chlorophyll content significantly increased by 33% at 100 µM Pb, while remaining steady at 50 and 200 µM displaying a hormesis effect. SS, TFC, and MDA levels showed no significant changes, whereas TPC trended upward at 200 µM. CAR levels were slightly elevated at 100 µM (37%) and exhibited a hormesis effect across Pb concentrations. Antioxidant enzyme activity generally increased, except for SOD, which consistently decreased in all Pb-treated groups. These findings suggest that *P. tremula* responds positively to Pb contamination at these levels.

Furthermore, the elemental analysis of P, K, Ca, Mg, S, Fe, Mn, Cu, and Zn in *P. tremula* leaves and roots clearly indicates that HM contamination in the nutrient medium leads to significant metabolic shifts distinct in the leaves and roots. In all cases, both positive and negative shifts can be noted (**Table 3**). In the leaves, As exposure shows a significant increase in S and Zn, while most other elements decrease, especially Fe and Mn. In the roots, P, K, Mg, Mn and S are elevated, while Fe, Cu, Ca and Zn are substantially reduced. At low concentrations (50 µM), Pb generally increases P in the leaves and roots and Zn in the leaves, decreases Fe in the leaves and Zn in the roots. As the concentration increases (100 µM and 200 µM), the trend continues with increases in P in the leaves with decreases of S, Mn, Zn in the leaves. Increases of K, Ca and Mg and decreases of Cu and Zn in the roots can be noted, while other elements show more variability. Cd exposure shows a mix of increases and decreases in the leaves and roots. In leaves, S and Zn increase with increasing concentration, with notable reductions in K, Mg, Mn, and Fe. In the roots, P, and Mg show large increases, while Mn and Zn decrease with increasing concentration. As is particularly toxic to both the leaves and roots, showing marked decreases in essential elements such as Fe, Mn, and Cu, but large increases in S and Zn.

**Table 3:** Concentrations of various elements (P, K, Ca, Mg, S, Fe, Mn, Cu, Zn) in the leaves and roots of *Populus tremula* in different experimental groups (from 3 pooled replicates; treated with 100 µM of As, 5, 25, 50 µM of Cd, and 50, 100 and 200 µM of Pb) expressed as percent differences from control – untreated group.

Leaves	P	K	Ca	Mg	S	Fe	Mn	Cu	Zn
As100	-1.85	4.83	-8.39	-9.07	61.40	-49.11	-27.99	-0.48	97.32
Pb50	23.96	8.54	4.94	6.63	4.57	-31.76	0.53	5.26	17.51
Pb100	20.51	-4.37	-17.23	-2.91	-12.71	6.47	-16.90	24.41	-13.48
Pb200	26.91	-4.71	11.62	35.90	-11.08	-18.19	-31.96	-31.16	-93.31
Cd5	16.92	-13.14	-9.17	-3.53	4.05	-6.17	-25.64	15.04	13.19
Cd25	-33.36	-24.89	-19.87	-20.61	15.76	-61.71	-13.53	-11.53	90.44
Cd50	-13.17	-30.87	1.10	-5.54	65.46	-45.60	-63.09	28.99	441.36
Roots									
As100	51.99	19.85	-26.29	21.06	52.92	-76.02	59.40	-38.74	-34.72
Pb50	15.83	9.00	-0.61	6.51	3.81	2.17	-5.97	3.73	-50.24
Pb100	26.83	30.56	15.23	74.02	16.41	79.41	58.54	-28.40	-43.44
Pb200	-20.27	9.57	10.17	57.57	-3.63	90.58	51.21	-35.19	-51.98
Cd5	58.61	16.80	1.39	73.89	-0.92	-5.74	-8.44	-16.29	-63.65
Cd25	306.66	27.18	0.36	77.41	22.61	19.73	-43.13	42.92	-24.36
Cd50	230.59	-9.78	-6.96	22.43	-8.55	-22.14	-88.67	34.41	-78.01

Overall, Pb has a more positive effect on element uptake in both leaves and roots at lower concentrations (50 µM), but higher concentrations (100 µM and 200 µM) show more complex interactions in the leaves, yet with continued increased metal concentrations (e.g., Fe, K, Mg) in the roots. Cd displays a similar complex trend, with some elements (like Zn and S) showing considerable increases at higher concentrations in the leaves, while others (like K in the leaves and Mn in the roots) decrease significantly.

### 3.2 HM impact on *Salix caprea*

The severe chlorosis observed in *P. tremula* treated with 500 and 1000 µM of As, along with its strong positive response to Pb treatment, prompted an adjustment in the tested concentrations for *S. caprea*. As a result, *S. caprea* exposed to 25, 50, and 100 µM of As exhibited improved TIs, ranging from 91% to 108% (**Table 4**). In these groups, TF varied between 0.02 and 0.11, while the leaf BCF ranged from 1.28 to 15.6, and the root BCF spanned from 65.33 to 172.72. Comprehensive data on HM concentrations in *Salix* seedlings can be found in **Table 4**. As levels ranged from 10 to 29 µg/g DW in the leaves and 323 to 490 µg/g DW in the roots. Cd concentrations were 58 to 484 µg/g DW in the leaves and 80 to 622 µg/g DW in the roots, while Pb levels varied from 22 to 92 µg/g DW in the leaves and 500 to 5372 µg/g DW in the roots. These results follow a similar trend observed in *Populus* seedlings, where increasing HM concentrations in the medium corresponded to higher accumulation in plant tissues, with roots consistently exhibiting greater HM levels than leaves.

**Table 4:** Bioconcentration factor (BCF), tolerance index (TI), translocation factor (TF) and heavy metal concentrations in the leaves and roots of *Salix caprea* in different experimental groups (from 3 pooled replicates; control – untreated, treated with 25, 50, 100 µM of As, 5, 25, 50 µM of Cd, and 200, 400 and 600 µM of Pb).

HM concentration in medium, µM		BCF		TI (%)	TF	Concentration in biomass, µg/g DW	
		Leaves	Root			Leaves	Roots
As	25	15.6	172.72	94.49	0.11	29.17 ± 1.16	322.99 ± 33
	50	5.49	107.45	107.61	0.04	20.58 ± 0.51	402.95 ± 137.42
	100	1.28	65.33	90.58	0.02	9.57 ± 0.81	489.99 ± 76.37
Cd	5	107.21	147.63	83.65	0.77	57.82 ± 14.12	79.62 ± 29.95
	25	132.26	211.52	48.87	0.62	356.65 ± 61.46	570.37 ± 39.19
	50	89.7	115.37	37.38	0.8	483.76 ± 73.79	622.22 ± 66.09
Pb	200	0.53	12.06	113.16	0.04	22.17 ± 1.92	499.98 ± 42.77
	400	0.75	48.91	98.8	0.01	62.05 ± 28.2	4056.83 ± 523.89
	600	0.74	43.18	82.48	0.02	91.84 ± 27.44	5371.73 ± 920.16

**Figure 4:**
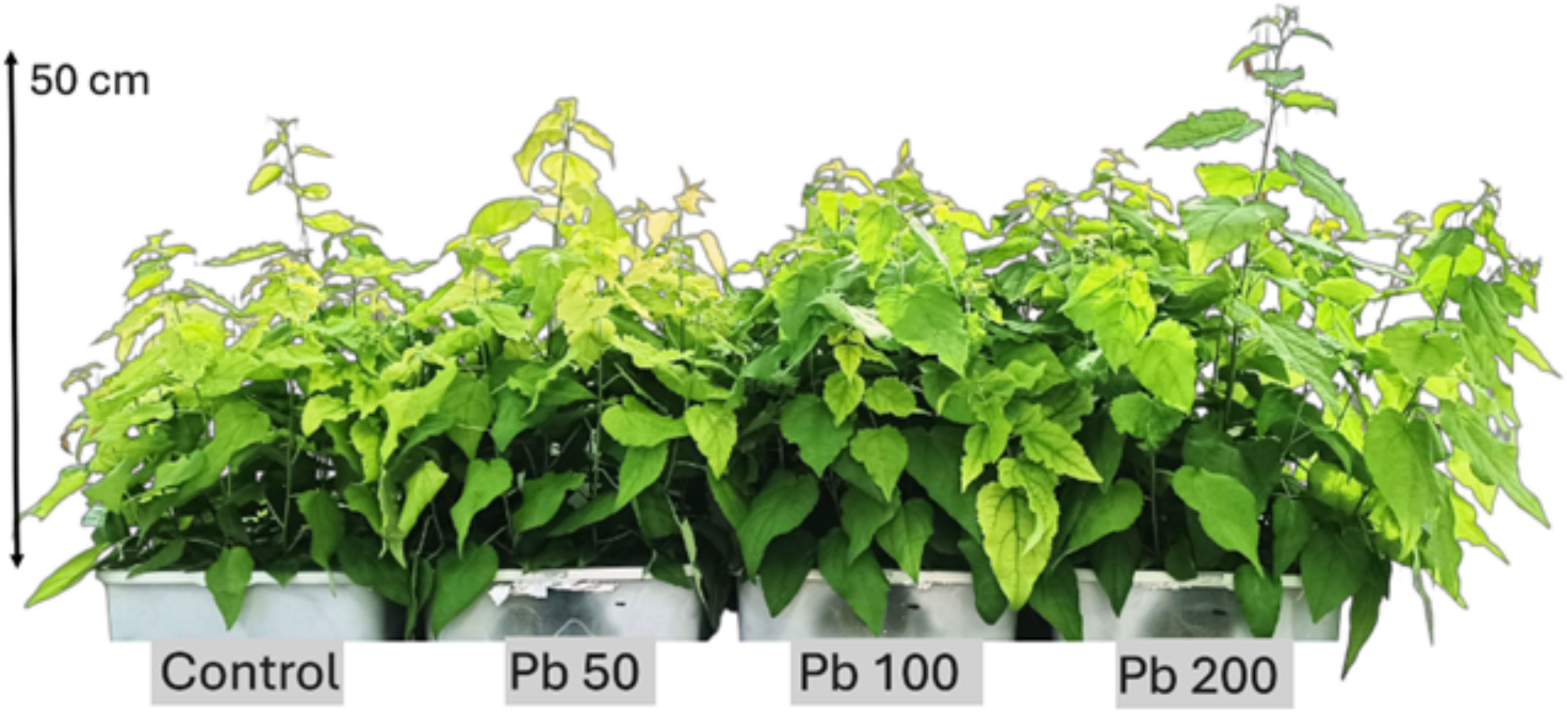
*Populus tremula* L. after 4 weeks grown in hydroponic conditions in different experimental groups (control – untreated, treated with 50, 100 and 200 µM of Pb).

Cd contamination yielded intriguing results (**Table 4**). *Salix* seedlings treated with Cd displayed significantly higher translocation factors compared to other tested groups, ranging from 0.62 to 0.8. TIs varied between 37% and 84% (**Figure 4**, **Table 4**), while the leaf BCF ranged from 89.7 to 132.26, and the root BCF spanned from 115.37 to 211.52. Pb treatment yielded TIs of 82-133%, TFs of 0.01-0.04, leaf BCF of below 1 (0.53-0.75) and root BCFs ranging from 12.06 to 48.91. Notably, all Cd concentrations resulted in a measurable reduction in growth. However, data from the lowest tested concentration (5 µM) indicate that *S. caprea* exhibits hyperaccumulator potential for Cd.

**Figure 5:**
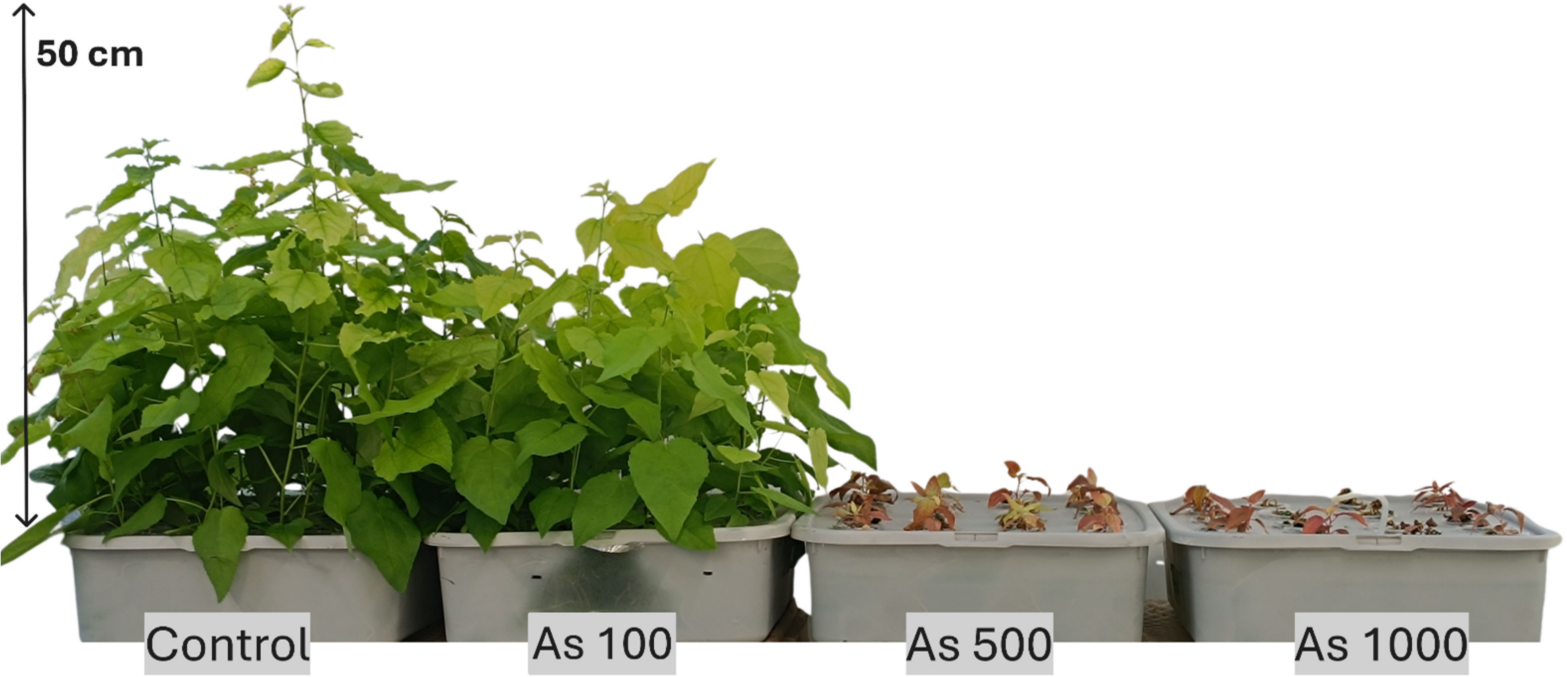
*Populus tremula* L. after 4 weeks grown in hydroponic conditions in different experimental groups (control – untreated, treated with 100, 500 and 1000 µM of As).

**Figure 6:**
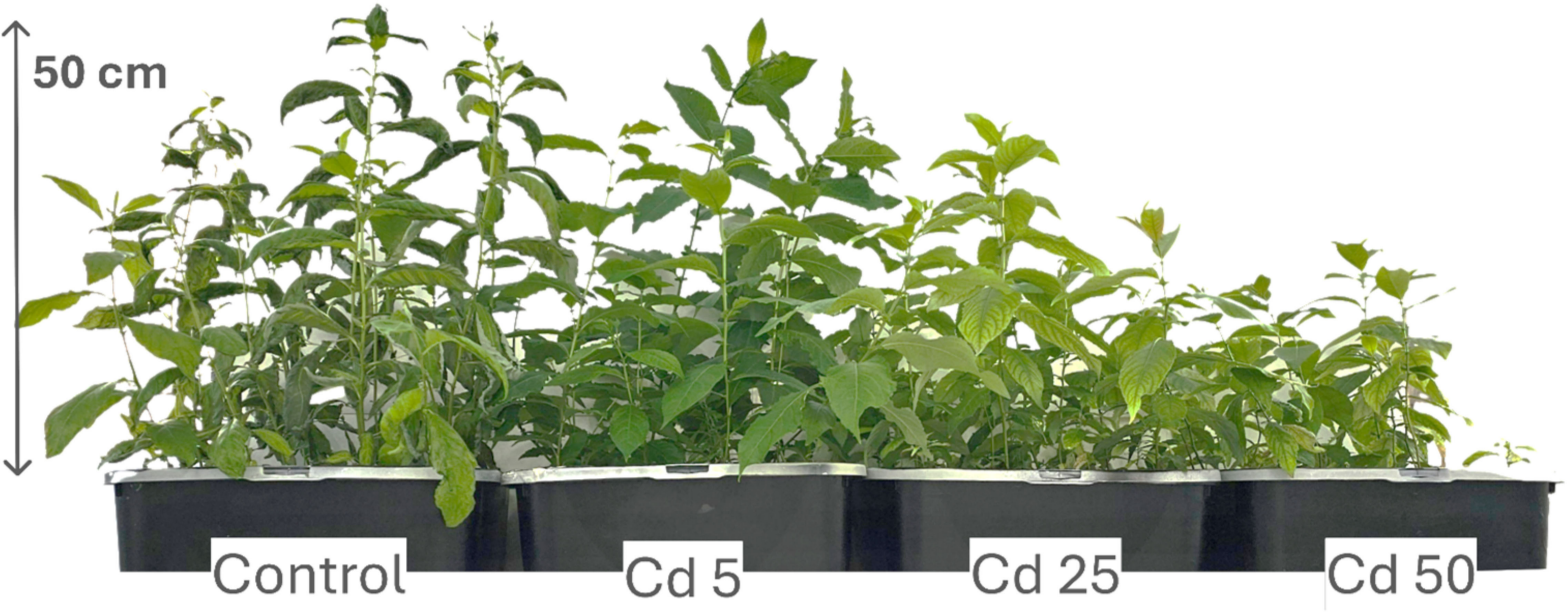
*Salix caprea* after 4 weeks grown in hydroponic conditions in different experimental groups (control – untreated, treated with 5, 25 and 50 µM of Cd).

Further analysis of *S. caprea* seedling biochemical health and growth markers reveals that the higher As concentrations (50 and 100 µM) induce significant shifts in the photosynthetic apparatus (**Table 5**, **Figures 5**, **6**). Total chlorophyll levels decline across all As-treated groups by 35–62% (**Figure 5**). Similarly, carotenoid levels initially decrease before increasing, with a notable 35% rise at 50 µM of As (**Table 5**, **Figure 6**). Growth, MDA, SS, antioxidant enzyme activity, TFC, and TPC levels remain relatively stable at the lower concentrations (25 and 50 µM). However, at 100 µM of As, antioxidant enzyme activity decreases across all tested enzymes except for SOD.

**Table 5:** Biochemical marker [total chlorophyll, carotenoid (CAR), total phenol (TPC) and total flavonoid (TFC) content, malondialdehyde (MDA), soluble sugar, (SS), antioxidant enzyme (catalase (CAT), superoxide dismutase (SOD), guaiacol peroxidase (POX), ascorbate peroxidase (APX), glutathione reductase (GR), glutathione S-transferase (GST)] levels in the leaves of *Salix caprea* in different experimental groups (control – untreated, treated with 25, 50, 100 µM of As, 5, 25, 50 µM of Cd, and 200, 400 and 600 µM of Pb).

HM concentration in medium, µM	Control	As			Cd			Pb		
	0	25	50	100	5	25	50	200	400	600
TPC	0.24 ± 0.02a	0.27 ± 0.01a	0.23 ± 0.02ab	0.19 ± 0.02b	0.21 ± 0.03ab	0.18 ± 0.03b	0.24 ± 0.02a	0.24 ± 0.01a	0.21 ± 0.03a	0.21 ± 0.01a
TFC	0.25 ± 0.05ab	0.21 ± 0.04a	0.24 ± 0.08a	0.25 ± 0.03a	0.3 ± 0.06a	0.16 ± 0.04b	0.17 ± 0.02b	0.31 ± 0.03a	0.35 ± 0.06a	0.29 ± 0.02a
SS	0.91 ± 0.15a	0.85 ± 0.11a	1.01 ± 0.14a	0.78 ± 0.11a	0.77 ± 0.11a	0.75 ± 0.13a	0.83 ± 0.06a	0.83 ± 0.13a	0.86 ± 0.14a	0.84 ± 0.09a
MDA	74.2 ± 4.58a	70.5 ± 6.06a	70.37 ± 2.66a	67.48 ± 4.71a	69.28 ± 3.71ab	68.85 ± 4.06ab	59.88 ± 4.73b	75.54 ± 4.31a	73.02 ± 4.94a	79.14 ± 4.71a
Total chlorophyll	2.85 ± 0.15a	1.09 ± 0.14b	1.84 ± 0.21ab	1.75 ± 0.33ab	4.76 ± 0.34b	1.76 ± 0.56a	1.8 ± 0.21a	4.07 ± 0.36ab	4.37 ± 0.54ab	4.59 ± 0.36b
Carotenoid content	326.64 ± 46.33 ac	287.27 ± 59.36a	440.25 ± 63.59b	394.71 ± 58.31bc	477.32 ± 29.7b	187.54 ± 76.49a	213.75 ± 41.47a	430.81 ± 15.77ab	482.63 ± 36.42b	471.15 ± 28.9b
Shoot height increase	254.15 ± 95.87ac	240.15 ± 79.17a	273.5 ± 45.83a	230.2 ± 107.22a	212.6 ± 105.01a	124.21 ± 83.76b	95 ± 64.06b	287.6 ± 106.77a	251.11 ± 110.9ac	209.61 ± 90.55c
Root length	448.8 ± 133.11a	446.2 ± 114.09a	480.5 ± 83.12a	425.6 ± 109.04a	341.25 ± 144.98b	327.65 ± 162.66b	304 ± 147.3b	396.5 ± 98.21ac	326.45 ± 125.72c	239.35 ± 111.96b
CAT	9.73 ± 0.77ac	9.37 ± 0.58bc	9.86 ± 1.03b	7.75 ± 0.36b	8.83 ± 0.39b	8.74 ± 0.35b	8.57 ± 0.46b	8.2 ± 0.46a	8.75 ± 0.46a	9.47 ± 0.63a
APX	100.94 ± 11.52a	95.16 ± 12.64a	98.95 ± 10.3a	101.75 ± 3.83a	117.87 ± 5.33b	109.77 ± 3.68b	109.97 ± 6.69ab	89.04 ± 4.45b	95.46 ± 3.52ab	101.22 ± 7.36a
POX	4.02 ± 0.33ac	5.35 ± 1.1a	3.58 ± 0.39c	2.79 ± 0.24b	3.63 ± 0.32ab	3.05 ± 0.14bd	2.64 ± 0.24d	3.38 ± 0.25b	4.03 ± 0.48ab	5.08 ± 0.5a
GR	61.05 ± 5.46a	55.34 ± 3.46a	61.54 ± 7.65a	43.17 ± 1.72b	50.54 ± 2.44b	47 ± 1.76b	48.43 ± 4.67b	46.15 ± 2.21b	49.91 ± 1.92bc	53.31 ± 2.62ac
GST	30.73 ± 2.49a	28.33 ± 2.08a	31.8 ± 4.89a	24.01 ± 1.02b	28.49 ± 1.38ac	26.27 ± 1.07b	26.53 ± 1.21bc	25.62 ± 2.32b	27.73 ± 2.7bc	28.21 ± 1.83ac
SOD	140.59 ± 46.8a	161.23 ± 61.95a	159.61 ± 47.45a	193.51 ± 65.96a	256.29 ± 114.57a	144.71 ± 57.52a	230.5 ± 124.33a	186.64 ± 60.29a	140.54 ± 46.03a	177.95 ± 66.47a

Different letters in the same row for one metal and the control denote a significant difference between concentrations based on Dunn’s test (*p*<0.05).

In contrast, the lowest Cd concentration (5 µM) led to a significant increase in both total chlorophyll concentration and carotenoid levels, rising by 67% and 46%, respectively. However, higher Cd concentrations resulted in declines for both parameters (**Figures 5**, **6**, **Table 5**). MDA and SS levels remained largely stable, but growth was negatively affected, particularly in the roots, with increasing Cd concentrations. Root length decreased by 24–32%, while shoot height increase was reduced by 16–63% (**Figure 4**). Antioxidant enzyme activity declined at the higher Cd concentrations (25 and 50 µM), except for SOD, which remained unchanged, and APX, which increased. At 5 µM Cd, only CAT, APX, and GR were affected, with APX activity rising by 17%, while CAT and GR levels decreased by 9% and 17%, respectively. Cd-treated seedlings also exhibited variability in both TPC and TFC.

In the Pb-treated group, growth parameters remained stable at the lowest concentration (200 µM) but declined at the higher concentrations (400 and 600 µM) (**Table 5**). Pb-treated seedlings also exhibited increased total chlorophyll levels, with a 61% rise at the highest concentration (**Figure 5**), along with elevated carotenoid levels (ranging from 32% to 44%) (**Figure 6**). TPC, SS, MDA, and SOD enzyme levels remained relatively unchanged, while TFC increased by 15-39%. At 200 µM Pb, most antioxidant enzyme activities were reduced, whereas at 400 µM, they exhibited some variability. Overall, these findings suggest that *S. caprea* has potential as a Cd hyperaccumulator at low concentrations. Additionally, this representative of the Salicaceae family, along with *P. tremula*, may be suitable for Pb phytostabilization.

Nutrient homeostasis plays a critical role in plant stress responses under HM exposure. Each metal demonstrates distinct patterns of uptake and distribution of nutrients across the *Salix* plant, with the roots and leaves responding differently based on the concentration of contamination (**Table 6**). As-treated groups exhibited a severe Ca and Mg depletion across roots and leaves, P depletion in the leaves, and Fe, Mn, Cu and Zn depletion in the roots. The declines in root nutrients were more pronounced, while leaves showed sporadic increases in metals. S accumulation was high, especially in roots. Fe, Mn and Cu showed elevated levels in the leaves. Pb-treated groups displayed a steady depletion of Ca and Mg in both roots and leaves. Zn accumulated in leaves but decreased in roots. Roots were less affected in P and K levels compared to leaves. Cu levels increased in the leaves, but decreased in the roots, while the other element response to Pb was variable. Under Cd contamination Cu and Zn levels were high in leaves but severely depleted in roots. The seedlings showed nutrient stress, with a general depletion of Ca, Mg, Fe, and Zn. Cd-treated roots accumulated P, K and S but showed losses in other nutrients.

**Table 6:** Concentrations of various elements (P, K, Ca, Mg, S, Fe, Mn, Cu, Zn) in the leaves and roots of *Salix caprea* in different experimental groups (from 3 pooled replicates; treated with 25, 50, 100 µM of As, 5, 25, 50 µM of Cd, and 200, 400 and 600 µM of Pb) expressed as percent differences from control – untreated group.

Leaves	P	K	Ca	Mg	S	Fe	Mn	Cu	Zn
As25	-42.81	6.05	25.15	19.23	348.72	5.94	25.42	157.09	792.16
As50	-31.31	-1.59	-22.74	-20.94	96.97	–	46.19	113.00	138.58
As100	-48.72	-14.32	-40.58	-37.14	64.83	147.04	24.26	131.26	-59.41
Pb200	-35.33	-4.51	-1.06	-8.35	3.65	19.89	71.81	54.44	423.76
Pb400	-30.94	-24.18	-25.00	-28.73	-32.34	-15.93	6.14	15.94	379.54
Pb600	-42.36	-30.07	-27.00	-27.46	-25.59	54.24	-1.50	17.67	185.69
Cd5	-40.62	-13.52	-25.20	-35.24	15.67	18.46	27.87	146.43	-7.60
Cd25	-55.81	1.92	-29.24	-36.15	77.99	-44.35	-19.47	168.57	457.21
Cd50	-61.88	-17.34	-47.39	-50.02	-1.43	-23.69	-36.92	215.35	639.30
Roots									
As25	-19.77	7.16	-51.92	-45.68	270.42	-63.33	-61.42	-45.57	-42.76
As50	19.67	16.86	-32.45	-23.02	313.82	-29.59	-23.64	-37.02	-20.04
As100	40.98	33.40	-43.55	-35.77	438.16	-44.10	0.81	-19.68	9.32
Pb200	3.35	-2.78	-39.68	-26.92	-15.69	-32.85	-23.59	-21.27	-28.22
Pb400	15.94	19.77	-1.48	16.19	12.83	23.93	32.87	-40.19	-25.22
Pb600	20.87	11.17	-35.66	-18.46	-11.04	-20.84	-9.40	-57.30	-52.13
Cd5	120.00	70.30	-12.33	-25.88	131.43	-31.19	-11.79	-74.22	-76.04
Cd25	45.94	42.78	-54.51	-44.45	149.71	-37.39	-54.35	-43.38	-54.04
Cd50	42.25	21.50	-22.51	-12.50	184.88	7.64	-12.29	-2.86	-33.29

Overall, Cd caused severe nutrient disruptions while Pb had a more moderate effect, allowing some Zn accumulation in leaves. Roots were more negatively affected across all metals.

## 4 Discussion

This study provides new insights into the phytoremediation potential of *Populus tremula* and *Salix caprea*, with a particular focus on seedling responses to As, Cd, and Pb exposure in a hydroponic system. Our findings demonstrate that both species exhibit distinct metal-specific and dose-specific responses during early development, with several key discoveries emerging from this research: (I) integrated assessment of antioxidant enzyme systems, secondary metabolites, and nutrient dynamics revealed species-specific tolerance mechanisms, and (II) species-specific bioaccumulation and translocation patterns were identified that inform targeted phytoremediation strategies. By analyzing growth, HM uptake efficiency, biochemical status, stress markers, and elemental composition shifts, this study offers a comprehensive evaluation of how these species respond to HM contamination at the critical seedling establishment phase. The results provide essential baseline data for understanding early-stage metal tolerance mechanisms and support the development of more targeted phytoremediation approaches using these economically and ecologically important species.

Most phytoremediation studies have focused on the ability of *P. tremula* and *S. caprea* to accumulate HMs when propagated from cuttings and/or grown in soil (Konlechner et al., 2013; Salam et al., 2022). In contrast, our study utilized seedlings and a hydroponic approach, eliminating soil-related confounding factors to allow precise quantification of metal uptake and physiological responses. This methodological choice offers a clearer understanding of metal stress adaptation at the early seedling stage and highlights previously underexplored biochemical and metabolic adjustments (Pietrini et al., 2010). Building upon these methodological advantages, our findings revealed distinct metal- and dose-specific responses that highlight the complexity of phytoremediation mechanisms. Pb exposure had minimal effects on growth and often induced hormesis, suggesting that low to moderate Pb levels may not severely impair physiological function in these species and in fact both species exhibited higher TI (based on shoot growth) ranging from 113% (50 µM) to 127% (200 µM) for *P. tremula* and 113% for *S. caprea* (200 µM of Pb). Conversely, Cd and As reduced TIs, and both shoot and root growth. These findings correspond with previous data, that Pb is less toxic to plants than Cd or As, possibly due to relatively reduced Pb mobility in the plant (Reddy and Patrick, 1977; Fritz et al., 2022). Furthermore, the observed hormetic effect has also been reported in previous studies on HMs. For instance, the potential Cd hyperaccumulator *Bidens pilosa* L. exhibited a 13.8% increase in shoot biomass (Sun et al., 2009), while Pb was found to enhance root system length in wheat (Erofeeva, 2014). Similar effects were later confirmed in corn for both Cd and Pb (Małkowski et al., 2020). However, growth reduction remains a widely documented response to HM exposure in plants (Schützendübel et al., 2001; Pietrini et al., 2010; Nahvi et al., 2024).

Complementing these growth responses, photosynthetic pigment analysis revealed further evidence of metal-specific adaptation mechanisms. Both Pb-treated trees showcased an increase in total chlorophyll levels and *S. caprea* also exhibited higher chlorophyll levels in seedlings treated with the lowest concentration of Cd (5 µM), while other Cd- and As-treated groups exhibited lower concentrations with carotenoid levels generally expressing the same tendencies. The increase in pigment content, specifically chlorophyll levels, has been previously observed, though not often, in other species (Agathokleous et al., 2020; Małkowski et al., 2020) with several potential mechanistic explanations behind this. This could be the cumulative result of a robust antioxidant system, which can mitigate oxidative stress and protect chlorophyll from degradation and an upregulation of biosynthetic pathways. For example, some HMs at low concentrations could potentially act as micronutrients [e.g., Cd mimicking Zn (Pongrac et al., 2009; Muszyńska and Labudda, 2019)]. While this is not very common for the non-essential elements like Cd, As and Pb, as opposed to higher doses of essential elements like Fe, Zn, etc., studies do mention the phenomenon (Muszyńska and Labudda, 2019). Some HMs can also induce phytohormone-like effects. This trait is not only linked with stimulating photosynthetic pigment production but may be responsible for slight lean toward shoot proliferation and simultaneously reduced root elongation, which was noted in Pb-treated groups in the current study. This was previously observed in corn treated with Pb. The authors discovered that the hormetic stimulation of shoot growth by HMs was consistently associated with increased auxin and flavonol levels, while maintaining hydrogen peroxide concentrations comparable to those in control plants (Małkowski et al., 2020).

On the other hand, HM toxicity often has a detrimental effect on the photosynthetic apparatus through a variety of pathways, affecting multiple components at different levels (Pietrini et al., 2010; Nahvi et al., 2024). Toxicity increases reactive oxygen species (ROS) and this can lead to peroxidation of thylakoid membranes, reducing electron transport efficiency (Bashir et al., 2015; Małkowski et al., 2020). HMs can directly inhibit crucial enzymes (like ALA dehydratase (Stobart et al., 1985; Sarangthem et al., 2011) and protochlorophyllide reductase (Stobart et al., 1985)), bind to key proteins (like D1) (Geiken et al., 1998) within the photosynthetic pathways. Furthermore, inhibition of oxygen-evolving complex disrupts water photolysis, reducing O_2_ evolution and ATP synthesis (Azmat et al., 2022). HMs can induce stomatal closure, reducing CO_2_ uptake and directly affecting RuBisCO activity too (Perfus-Barbeoch et al., 2002; Wang et al., 2009). This was shown in a hydroponic experiment with Cd-treated *Arabidopsis thaliana* L., *Vicia faba* L. and *Commelina communis* L (Perfus-Barbeoch et al., 2002). Pb can affect PEP carboxylase, and other key enzymes lowering CO_2_ assimilation (Wang et al., 2009), while As can interfere with phosphate metabolism, limiting ATP production (Finnegan and Chen, 2012).

To understand the mechanisms underlying these photosynthetic responses, we conducted detailed biochemical profiling that represents a major strength of the current study, which offers mechanistic insights into the antioxidant responses and metabolic shifts induced by HMs. The use of multiple biochemical markers, including lipid peroxidation (MDA), soluble sugars, flavonoids, and phenolic compounds, provides a holistic view of the stress response mechanisms in the tested species. Interestingly, in the current study while growth and pigment concentrations were affected, HM treatment minimally altered biochemical markers such as MDA and SS. In both trees, these indicators remained relatively steady, with the exception being Cd-treated *Populus* showing elevated MDA at the lowest concentration and upward trending SS levels. As MDA directly indicates lipid peroxidation levels, it gives a clear image of the HM caused ROS damage (Sun et al., 2009; Erofeeva, 2014). However, if antioxidant defences are highly active, lipid peroxidation (and thus MDA levels) may remain low despite HM exposure (Pang et al., 2002). In contrast SS levels can indicate several things; a direct increase in glucose production – carbon metabolism, and osmotic adjustment, which relates to stress levels (Rosa et al., 2009). Also, instead of accumulating soluble sugars, the plant might be diverting carbon into secondary metabolites (e.g. phenols) to enhance the antioxidative response (Caretto et al., 2015). Increased sugar transport to roots may also prevent SS buildup in leaves (Du et al., 2020).

As in both cases of HM impact on the photosynthesis system, and stress indicator levels, ROS plays a huge role, it’s imperative to look into the enzymatic and non-enzymatic antioxidant systems, i.e., secondary metabolites (TPC, TFC) and antioxidant enzymes (SOD, CAT, POX, APX, GR, GST) (Asare et al., 2023). These two systems work together but differ in their mechanism, speed, energy cost, and efficiency. Their deployment depends on the type, intensity, and duration of stress. Overall, the enzymes represent the first line of defense, i.e., rapid response, lower cost, while the non-enzymatic antioxidants represent the secondary or long-term defense (higher cost), which is triggered when ROS levels persist, or enzymatic defenses are insufficient (Gill and Tuteja, 2010). The slightly elevated MDA and SS levels noted in the *Populus* trial (Cd at 5 µM) can be attributed to induced stress, however, MDA levels decreased in the two higher concentration groups. This could be the result of the accompanying impact of slightly elevated SS and increased TPC (33-53%) levels. Furthermore, the decrease in antioxidant enzyme levels is evident in the same group. This further showcases the shift towards SMs rather than enzymatic response, which mitigated lipid damage. A similar response was noted in Cd-treated *Pinus sylvestris* L. Initially the treatment caused an increase in the enzymatic response, but it later shifted towards the accumulation of soluble phenolics (Schützendübel et al., 2001). Opposingly, *P. tremula* group treated with 50 µM of Cd demonstrates an increase in enzymatic activity alongside elevated TPC. The activation of both systems suggests an increase in stress levels. Again, this was previously noted in trials with other HMs in maize (AbdElgawad et al., 2020).

Cd-treated *S. caprea* showcases yet another response variation – reduced enzyme production (except for steady SOD and slightly upregulated APX) and reduced TFC production (33-35%) in the two higher Cd concentrations, and reduced TPC in the 25 µM treated group. At the same time growth rates were declining, as were photosynthesis pigment content. This indicates severe oxidative stress and metabolic disruption. Cd and Pb toxicity is known to interfere with phenylalanine ammonia-lyase (PAL), a key enzyme in flavonoid biosynthesis, in both a positive and negative way (Jańczak-Pieniążek et al., 2023). Additionally, Cd might have damaged chloroplasts and plastids (Lee and Back, 2017), which can impact phenol synthesis, or flavonoids and phenolics could have been depleted too quickly due to high ROS levels, exceeding the plant’s capacity to replenish them. Cd may also inhibit key enzyme production directly through cofactor replacement, protein binding or glutathione metabolism (Stobart et al., 1985; Geiken et al., 1998; Paradiso et al., 2008; Sarangthem et al., 2011).

A similar response can be noted in As-treated *S. caprea*. The group treated with 100 µM was mainly affected with reduced levels of CAT, POX, GR, GST and TPC. However, as the growth rate is similar to the control group, it could mean that *S. caprea* relies less on enzymatic detoxification and more on metal exclusion. The plant might be sequestering As into vacuoles or binding it to cell wall components (Rascio and Navari-Izzo, 2011). HM exclusion strategy is also a high likelihood as HM accumulation data suggests that the plant may be blocking As uptake at the root level (high levels in the roots vs. the leaves), reducing the need for detoxification compounds. Moreover, As is known to interfere with enzyme activity by binding to thiol groups (-SH) in proteins (January et al., 2008), as does Cd (Stobart et al., 1985; January et al., 2008), possibly leading to inhibited GR and GST (which require thiol-based glutathione to function) (Gill and Tuteja, 2010). Overall, this indicates that *S. caprea* employs As avoidance strategies at the concentrations tested rather than detoxification. Furthermore, elevated antioxidant enzyme activity (except for SOD) suggests an active detoxification mechanism that mitigates oxidative damage in *Populus* treated with As (100 µM). This suggests H_2_O_2_ accumulation and increased reliance on the ascorbate-glutathione (ASC-GSH) cycle to mitigate oxidative stress and As toxicity. This was previously observed in wheat with Cd toxicity, whereby ASC-GSH cycle enzymes showed increased activity in roots, correlating with Cd accumulation. Supplementing ASC biosynthesis alleviated Cd-induced oxidative stress in roots, without affecting Cd uptake (Paradiso et al., 2008). These findings reiterate that the ASC-GSH cycle is playing a crucial role in systemic antioxidant defense.

Pb treated *S. caprea* also exhibits efficient enzymatic detoxification mechanism with the activities of 5 out of 6 enzymes elevated (except for SOD), with other parameters steady at 200 µM. This changes with increasing concentrations – the metabolic shift towards increased enzyme and carotenoid (carotenoids may act as ROS scavengers too (Gill and Tuteja, 2010)) production is evident. Moreover, at the lower Pb concentrations studied in *P. tremula*, an increase in the enzymatic response was observed, but no corresponding SM production or other stress indicators. This, in part, corresponds with a review on Pb impact on plant metabolism. Collin et al. note that Pb treatment impact antioxidant enzyme activity positively, but they don’t mention SMs (Collin et al., 2022).

These biochemical adaptations directly influence metal accumulation patterns, with HM concentrations in both species being more concentrated in the roots for all three metals tested. The highest concentrations in the roots were noted in *S. caprea* for all three metals (490, 622, and 5372 µg/mg DW respectively for As, Cd and Pb), while the highest concentrations in the leaves varied – Pb and As were also highest in *S. caprea* (29 and 92 µg/mg DW respectively for As and Pb), but Cd concentrations were highest in *P. tremula* (538 µg/mg DW). Generally, the concentrations of HMs in both leaves and roots were higher with the increasing HM concentrations in the substrate. These differences reinforce the idea that different genotype/species plays a significant role in determining a plant’s bioaccumulation potential.

BCFs in both vegetative organs varied, with root numbers being higher. A BCF>1 signifies efficient metal accumulation, supporting phytoextraction, whereas BCF<1 suggests exclusion or limited uptake, favoring phytostabilization. Pb BCFs were around 1 in the leaves in both species, while they ranged from 12 to 49 in *S. caprea* and 16 to 66 in *P. tremula* roots. Cd BCFs were 90–132 in *Salix* and 100–110 in *Populus* leaves, while they ranged from 115 to 212 in *S. caprea* and 318 to 361 in *P. tremula* roots. As BCFs were 1–16 in *Salix* and were ~2 in *Populus* leaves, while they ranged from 65 to 173 in *S. caprea* and were 280 in *P. tremula* roots. In general, the TFs in both species were low. A TF>1 indicates efficient HM translocation to shoots, favoring phytoextraction, whereas TF<1 suggests metal retention in the roots, making the plant more suitable for phytostabilization. For Pb they ranged from 0.01 to 0.06 in both species, for As they were 0.01-0.11. These TFs along with the BCFs indicate that both species demonstrate strong phytostabilization capacity for Pb, suggesting their potential use in contaminated environments. Phytostabilization is a remediation technique whereby plants reduce the mobility and bioavailability of contaminants in the substrate. This process helps prevent the spread of pollutants, thus minimizing their uptake by other plants or leaching. Various mechanisms can be employed by the plant to achieve this goal, however in the case of the current study root absorption and binding is the most likely. Plant roots absorb contaminants but do not translocate them to aerial parts, keeping them contained in the rhizosphere (Rascio and Navari-Izzo, 2011). Furthermore, on the subject of Pb hyperaccumulation, Egendorf et al. note that while universal standards for Pb hyperaccumulation are not set, up until 2020 no species was shown to have BCF and TF over 1 and accumulate 1 g/kg of Pb in the roots, i.e., 0.1% (Egendorf et al., 2020). The current data is in line with their assessment, however *S. caprea* did notably exhibit >0.5% accumulation rate in the roots, with a high BCF in Pb trials.

The TFs were higher for Cd, ranging 0.25-0.34 in *Populus* and with Cd-treated *S. caprea* displaying increased translocation potentials (TF 0.62-0.8) in *Salix*. This aligns with prior studies suggesting that *Salix* species possess strong Cd accumulation capabilities (Xu et al., 2019; Kong et al., 2020). Notably, plants with TF values >1 are considered hyperaccumulators, however even when TF values remain below 1, as in the case of *S. caprea* and Cd, the rapid growth and substantial biomass production of these trees could likely compensate for lower translocation efficiency, making them effective candidates for phytoremediation in the form of phytoextraction (Pulford and Watson, 2003). Unlike phytostabilization, phytoextraction focuses on long-term removal of contaminants by accumulating them in harvestable biomass. HMs are stored in vacuoles or bound to cell wall components in leaves and stems to minimize toxicity. Interestingly, some plants have adapted to sequester and detoxify HMs in cell components, which has been linked to reduced herbivory, pest resistance, and pathogen deterrence in plants.

The observed bioaccumulation patterns are intrinsically linked to broader physiological adjustments, as evidenced by the differential nutrient uptake patterns that suggest complex ion-homeostasis mechanisms. HMs can interfere with nutrient absorption at the root level, altering the availability and mobility of elements in plant tissues through competition for transporters or damage to the roots (Muszyńska and Labudda, 2019). A trend of reduced levels of Ca and Mg was noted in all experimental groups of tested metals in *S. caprea* in both leaves and roots. It has long been known that HMs can mimic Mg and thus take its place in the chlorophyll complex – this being one of the key mechanisms behind the damage to the photosynthesis apparatus by HMs (Riyazuddin et al., 2022). Additionally, Ca reduction with increasing Cd concentrations has been reported for barley (Veltrup, 1981). Similarly, nutrient leaching is also a likely scenario. For example, membrane lipid peroxidation causes K and Mg leakage from leaves. Murphy et al. reported that K leakage from *Arabidopsis* leaves did increase short term under Cu treatment (Murphy et al., 1999). In the current study the general trend was towards P, Ca, and Mg reduction in the leaves of both species, with the additional reduction of K in *Salix*. As mentioned, HMs can interfere with P assimilation (Finnegan and Chen, 2012). Additionally, oxidative stress can trigger Fe and Mn remobilization (noted in *P. tremula*), as both Fe and Mn are components in the antioxidative mechanism (Briat et al., 2010; Chandra et al., 2020). HMs may alter the redistribution and translocation patterns, i.e., xylem and phloem loading of nutrients, leading to imbalances in leaves and roots (Page and Feller, 2015). This was especially notable in both tested species in P and K patterns. Furthermore, “co-accumulation” is also a possibility, e.g., both Cd²^+^ and Zn²^+^ use the ZIP family transporters, and Zn accumulation was shown to positively correlate with increased Cd accumulation (Pongrac et al., 2009). In the current study, this phenomenon was noted in both species leaves with *Salix* exhibiting enhanced Zn levels of up to 639% and *Populus* expressing enhanced Zn levels of up to 441% (in both cases Zn levels and Cd levels increased with increasing concentrations of Cd). Moreover, S accumulation is a key protective strategy against HM toxicity (Dixit et al., 2016). In rice, high S treatment has been shown to reduce As accumulation in shoots by altering As transporters and enhancing antioxidant enzyme activities (Dixit et al., 2016). In the current study S accumulation was noted in both species in both leaves and roots.

Integrating our nutrient dynamics findings with the photosynthetic responses provides mechanistic insights into adaptive strategies. The relationship between nutrient levels and enhanced total chlorophyll levels in specific treatment groups reveals the intricate nature of metal tolerance mechanisms. For example, in Cd- and Pb-treated *Salix* (5 µM of Cd, 600 µM of Pb) and *Populus* (25 µM of Cd, 100 µM of Pb). In both cases of Cd, a steady increase in S levels was observed. Enhanced levels of Cu and Zn can be noted in Pb-treated *Salix* (600 µM), while P and Cu levels increased in *Populus* (100 µM of Pb). Various nutrients (P, K, Ca, Zn, Cu) were shown to enhance chlorophyll content by contributing indirectly by supporting photosynthesis, enzyme activity, and chlorophyll stability (Widjaja et al., 2024). These findings highlight the intricate interplay between nutrient dynamics and chlorophyll accumulation, suggesting a potential adaptive response to heavy metal exposure in *Salix* and *Populus*.

## 5 Conclusions

This study underscores the potential of *P. tremula* and *S. caprea* in phytoremediation applications, particularly for Pb phytostabilization (*P. tremula* and *S. caprea*) (concentrations equivalent up to 200 µM of Pb(NO_3_)_2_) and Cd phytoextraction (*S. caprea*) (concentrations equivalent up to 5 µM of CdSO_4_). At these levels a hormetic effect for both Pb-treated species is evident, while TF of 0.77 was noted in Cd-treated *S. caprea* (5 µM). A deep dive into the biochemical and physiological responses provides a clear understanding of how *P. tremula* and *S. caprea* react to heavy metal stress, highlighting the antioxidant response hierarchy, i.e., an initial enzymatic response, and a shift towards secondary metabolite production should the stressful conditions continue or are too severe, and shifts in elemental composition (Cd and Zn co-accumulation standing out). Future research should focus on long-term field studies to validate these findings in soil environments, explore microbial interactions that may enhance metal uptake and plant health, and assess strategies for biomass management post-harvest. Additionally, future research should focus on long-term effects, particularly concerning the sustainability of phytoremediation, which remain to be explored. Overall, this study contributes to a growing body of evidence supporting the use of fast-growing tree species in sustainable phytoremediation strategies, providing a foundation for further research into optimizing their efficiency for large-scale applications.

## Data Availability

The raw data supporting the conclusions of this article will be made available by the authors, without undue reservation.
