# Supplementary material for: Evaluating Populus tremula L. and Salix caprea L. for phytoremediation: growth, metal uptake, and biochemical responses under arsenic, cadmium, and lead stress
Source: Front Plant Sci. 2025 Aug 1;16:1617432. doi: 10.3389/fpls.2025.1617432 (PMC12354478; doi:10.3389/fpls.2025.1617432)
Supplement: Supplementary file 1 [file Table1.docx]

**Table S1.** Detailed list of the formulas for biochemical analyses and various indices used in the current study.

| Parameter | Formula | Significance |
| --- | --- | --- |
| Chlorophyll *a*, *b*, and total carotenoids (CAR) | C(chl *a*) = (13.36 × A_664_) − (5.19 × A_648_)  C(chl *b*) = (27.43 × A_648_) − (8.12 × A_664_)  C(carotenoids) = (1000 × A_471_ − 2.13 × C(chl *a*) − 97.64 × C(chl *b*))/209    where A is the absorption of the extract at the respective wavelength; C(chl *a*), C(chl *b*) and C(carotenoids)—concentrations of alpha and beta chlorophyll and total carotenoids in the extract (µg/mL).  Cx(µg/g)=(C×V×W)/M  where Cx is the concentration of pigments in fresh leaves biomass (μg/g); C—concentration of pigments in the extract (μg/mL); V—volume of crude extract (ml); W—dilution of crude extract (units); M—weight of extracted biomass (g). | Indicators of photosynthetic performance and plant vitality; reduction suggests photosynthetic inhibition due to metal stress. |
| Total phenolic content (TPC) | Calibration curve: y = 0.0241x + 0.0206 (R^2^ = 0.9912).  TPC is expressed as micrograms of gallic acid equivalent in one gram of fresh mass (mg/g):  Concentration (mg/g) = (C × V)/m  where C is the concentration obtained from the calibration curve (mg/mL); V—extract volume (ml); m—weight of fresh biomass extracted (g). | Reflects the level of phenolic secondary metabolites involved in non-enzymatic antioxidant defense against oxidative stress. |
| Total flavonoid content (TFC) | Calibration curve: y = 0.0366x + 0.0122 (R_2_ = 0.995).  TFC is expressed as micrograms of the quercetin equivalent in one gram of fresh biomass (mg/g):  Concentration (mg/g) = (C × V)/m  where C is the concentration obtained from the calibration curve (mg/mL); V—extract volume (ml); m—weight of raw biomass extracted (g). | Represents the content of flavonoids, which act as antioxidants and stress mitigators under heavy metal exposure. |
| Total protein | Calibration curve: y = 0.1003x − 0.0462 (R_2_ = 0.9747).  The total amount of protein is expressed as micrograms of the BSA equivalent in one milliliter of crude extract (mg/mL). It is calculated according to the following formula:  A (mg/mL) = ((C × V)/P)/1000  where A is total protein concentration (mg/mL crude extract); C—concentration calculated from the calibration curve (mg/g); V—extract volume (ml); P—weight of raw biomass extracted (g). | Indicates the overall metabolic activity; can decrease under stress or increase with stress-induced protein expression. |
| Catalase (CAT) | CAT activity (µmol H_2_O_2_/mg (protein) per min) = ((S_c_ × V_t_)/(0.478 × V_e_ × 39.4))/P_eq_  where S_c_—slope coefficient; V_t_—total sample volume; V_e_—total extract volume; P_eq_—BSA equivalent based on standard curve. | Enzymatic antioxidant that breaks down hydrogen peroxide, protecting cells from oxidative damage. |
| Ascorbate Peroxidase (APX) | APX activity (µmol ASC/mg (protein) per min) = ((S_c_ × V_t_)/(0.478 × V_e_ × 2.8))/P_eq_  where S_c_—slope coefficient; V_t_—total sample volume; —total extract volume; P_eq_—BSA equivalent based on standard curve. | Detoxifies hydrogen peroxide using ascorbate; a key player in the plant’s antioxidant defense system. |
| Guaiacol Peroxidase (POX) | POX activity (µmol oxidized pyrogallol/mg (protein) per min) = ((S_c_ × V_t_)/(0.478 × Ve × 2.46))/P_eq_  where S_c_—slope coefficient; V_t_—total sample volume; V_e_—total extract volume; P_eq_—BSA equivalent based on standard curve | Catalyzes the breakdown of hydrogen peroxide and reflects oxidative stress response. |
| Glutathione Reductase (GR) | GR activity (µmol NADPH/mg (protein) per min) = ((S_c_ × V_t_)/(0.478 × V_e_ × 6.22))/P_eq_  where S_c_—slope coefficient; V_t_—total sample volume; V_e_—total extract volume; P_eq_—BSA equivalent based on standard curve. | Maintains reduced glutathione (GSH), essential for redox balance and detoxification. |
| Glutathione S-transferase (GST) | GST activity (µmol NADPH/mg (protein) per min) = ((S_c_ × V_t_)/(0.478 × V_e_ × 6.22))/P_eq_  where S_c_—slope coefficient; V_t_—total sample volume; V_e_—total extract volume; P_eq_—BSA equivalent based on standard curve. | Catalyzes the conjugation of glutathione to toxic substances, aiding in detoxification of metals. |
| Superoxide dismutase (SOD) | SOD activity (µmol O_2_·-/mg (protein) per min) = (S_c_ /(50 × Ve × P_eq_)) × t  where S_c_—slope coefficient; P_eq_ — BSA equivalent based on standard curve; V_e_ — total extract volume; t — time (min). | Converts superoxide radicals into hydrogen peroxide and oxygen, initiating the ROS detox pathway. |
| Malondialdehyde content (MDA) | MDA (nmol g^-1^ FW) = 6.45 × (A_532_ – A_600_) – 0.56 × A_450_  where A_532_ is the absorption value at 532 nm wavelength; A_600_ is the absorption value at 600 nm wavelength; A_450_ is the absorption value at 450 nm wavelength. | A marker of lipid peroxidation and oxidative membrane damage; higher levels indicate stress severity. |
| Soluble sugars (SS) | SS (mg g^-1^ FW) = (((A_620_ – 0.13252)/0.0144)/m)1000  where A_620_ is the absorption value at 620 nm wavelength; m is the weight of extracted biomass (g) | Osmoprotectants that also contribute to ROS scavenging and energy supply during stress. |
| Bioconcentration factor (BCF) | BCF = C_plant_ / C_solution_  Where C_plant_ —  metal concentration in plant tissue (root or shoot) (mg/kg DW);  C_solution_ —  metal concentration in the growth medium (µM or mg/L) | Indicates the plant’s capacity to accumulate metals from the medium into its tissues. |
| Translocation factor (TF) | TF = C_shoot_ / C_root_  Where C_shoot_ — metal concentration in shoot (mg/kg DW)    C_root_ — metal concentration in root (mg/kg DW) | Describes the ability of a plant to move absorbed metals from roots to aboveground tissues. |
| Tolerance index (TI) | TI = Height_treatment_ / Height_control_ | Quantifies the plant's ability to maintain growth in contaminated conditions relative to control. |
